# Supplementary material for: Physical measures of physical functioning as prognostic factors in predicting outcomes for neck and thoracic pain: Protocol for a systematic review
Source: PLoS One. 2025 Jan 24;20(1):e0316827. doi: 10.1371/journal.pone.0316827 (PMC11760039; doi:10.1371/journal.pone.0316827)
Supplement: S2 File — This file shows MEDLINE search strategy. (DOCX) [file pone.0316827.s002.docx]

S2 file. MEDLINE (Ovid) search strategy

| 1 | Cervical Vertebrae/ or cervical vertebra*.mp. |
| --- | --- |
| 2 | exp Thoracic Vertebrae/ or thoracic vertebra*.mp. |
| 3 | ((cervical or thoracic or dorsal) adj spin*).tw,kf. |
| 4 | (upper back or mid*back or cervico*thoracic or neck).tw,kf. |
| 5 | pain.mp. or exp Pain/ |
| 6 | or/1-4 |
| 7 | 5 and 6 |
| 8 | neck pain.mp. or exp Neck Pain/ |
| 9 | neck*ache*.tw,kf. |
| 10 | cervicalgia.tw,kf. |
| 11 | cervicodynia.tw,kf. |
| 12 | (neck pain adj8 cervicogenic headache).tw,kf. |
| 13 | whiplash*.tw,kf. |
| 14 | exp Radiculopathy/ |
| 15 | radicul*.tw,kf. |
| 16 | (radicul* adj4 (neck or cervical or thoracic)).tw,kf. |
| 17 | spondylosis.mp. or exp Spondylosis/ |
| 18 | ((cervical or thoracic) adj4 (radiculopathy or spondylosis)).tw,kf. |
| 19 | ((non*specific or mechanical or idiopathic) adj (neck or thoracic) adj  pain).tw,kf. |
| 20 | or/7-19 |
| 21 | exp Prognosis/ |
| 22 | prognos*.tw. |
| 23 | predict*.tw. |
| 24 | exp Follow-Up Studies/ |
| 25 | incidence.tw. |

| 26 | exp Cohort Studies/ |
| --- | --- |
| 27 | Prospective Studies/ |
| 28 | Longitudinal Studies/ |
| 29 | course.tw. |
| 30 | ((cohort or prospective or longitudinal or incidence or follow-up) adj5 (study  or design or analys?s or data)).tw. |
| 31 | or/21-30 |
| 32 | outcome assessment.mp. or Outcome Assessment, Health Care/ |
| 33 | (outcome* adj3 measur*).tw,kf. |
| 34 | (physical adj5 (outcome* or measur* or assess* or test* or function*)).tw,kf. |
| 35 | (objective adj (outcome* or assess* or measur*)).mp. |
| 36 | ((impairment* or performance* or activit*) adj5 (measur* or test* or  assess*)).tw,kf. |
| 37 | ((clinical or physical or function*) adj4 outcome*).tw,kf. |
| 38 | physical functional performance.mp. or Physical Functional Performance/ |
| 39 | exp Physical Endurance/ or endurance.mp. |
| 40 | postural balance.mp. or exp Postural Balance/ |
| 41 | task performance and analysis.mp. or "Task Performance and Analysis"/ |
| 42 | exp Work Capacity Evaluation/ |
| 43 | (coordination adj3 (test* or measur* or assess*)).tw,kf. |
| 44 | range of motion.mp. or exp "Range of Motion, Articular"/ |
| 45 | movement.mp. or exp Movement/ |
| 46 | joint position error.tw,kf. |
| 47 | proprioception.mp. or exp Proprioception/ |
| 48 | muscle strength.mp. or exp Muscle Strength/ |
| 49 | muscle contraction.mp. or exp Muscle Contraction/ |
| 50 | isometric contraction.mp. or exp Isometric Contraction/ |
| 51 | ((isometric or isokinetic or isotonic) adj strength).tw,kf. |

| 52 | activities of daily living.mp. or "Activities of Daily Living"/ |
| --- | --- |
| 53 | Self Care/ or self-care.mp. |
| 54 | hand strength.mp. or Hand Strength/ |
| 55 | grip strength.tw,kf. |
| 56 | exp exercise test/ or exp walk test/ |
| 57 | exercise test.mp. or exp Exercise Test/ |
| 58 | walk test.mp. or Walk Test/ |
| 59 | exp Gait/ or gait.mp. |
| 60 | gait analysis.mp. or exp Gait Analysis/ |
| 61 | walking speed.mp. or Walking Speed/ or Walking/ |
| 62 | stair climbing.mp. or Stair Climbing/ |
| 63 | manual muscle test*.tw,kf. |
| 64 | deep neck flexor endurance test.tw. |
| 65 | cranio*cervical flexion test.tw. |
| 66 | (timed up and go test).tw,kf. |
| 67 | functional capacity evaluation.tw,kf. |
| 68 | baltimore therapeutic equipment work simulator II.tw. |
| 69 | Functional Impairment Test Hand and Neck/Shoulder/Arm (FIT-HaNSA).tw. |
| 70 | (step adj count*).tw,kf. |
| 71 | smart*watch.tw,kf. |
| 72 | exp Accelerometry/ or acceleromet*.mp. |
| 73 | (activit* adj5 monitor*).tw. |
| 74 | exp wearable electronic devices/ or exp fitness trackers/ |
| 75 | muscle strength dynamometer.mp. or Muscle Strength Dynamometer/ |
| 76 | (dynamomet* or pedomet* or inclinomet* or goniomet*).tw,kf. |
| 77 | stride length.tw,kf. |
| 78 | cadence.tw,kf. |
| 79 | (stair* adj3 climb*).tw. |

| 80 | (spatio*temporal adj3 gait).tw,kf. |
| --- | --- |
| 81 | ((stance or swing) adj phase).tw,kf. |
| 82 | ((single* or double*) adj limb support).tw. |
| 83 | or/32-82 |
| 84 | 20 and 31 and 83 |
